# Mindfulness in social media exposure: the pressure-reducing valve for fear of missing out and social media fatigue

**DOI:** 10.3389/fpsyg.2025.1641462

**Published:** 2025-09-05

**Authors:** Xiaoxiao Huang

**Affiliations:** School of Global Journalism and Communication, Southwest University of Political Science & Law, Chongqing, China

**Keywords:** mindfulness, social media exposure, perceived value, fear of missing out, social media fatigue

## Abstract

**Introduction:**

In the past decade, the fear of missing out (FoMO) has gained widespread attention as a prominent manifestation of the negative effects of social media. However, research on the causes, significance, and targeted methods for mitigating FoMO remains incomplete. To address this issue, this study developed an integrated model that incorporates social media exposure, perceived value, mindfulness, FoMO, and social media fatigue.

**Methods:**

Using the partial least squares (PLS) method, this study conducted an empirical analysis of 406 Chinese social media users.

**Results:**

Findings indicate that perceived value functions as an important antecedent of FoMO. Moreover, FoMO is not merely a conspicuous negative effect of social media exposure; it is also a key contributor to social media fatigue. Additionally, the negative associations of mindfulness with FoMO and social media fatigue underscore its effectiveness in alleviating both conditions.

**Discussion:**

Theoretically, this study demonstrates the pivotal mediating role of FoMO in the negative impacts of social media and highlights mindfulness as a promising protective factor within digital contexts. Practically, the results position mindfulness as a valuable resource for mitigating the adverse impacts of social media and fostering sustainable digital engagement.

## 1 Introduction

The rapid proliferation of social media has not only fundamentally reshaped individuals' media consumption habits and interpersonal interaction patterns but has also raised a host of psychological and behavioral concerns reflecting its adverse effects. These concerns encompass but are not limited to compulsive use, oversharing, anxiety, addiction, and problematic sleep (Thorisdottir et al., 2019; Sands et al., 2020; Dhir et al., 2021; Montag et al., 2024). Therefore, there is growing recognition of the importance of understanding the underlying psychological mechanisms behind these negative effects and exploring effective strategies to mitigate them.

Fear of missing out (FoMO) is recognized as a prominent daily manifestation of the possible adverse effects of social media, playing a crucial role in our understanding of its negative consequences (Tandon et al., 2021b). FoMO, defined as “a pervasive apprehension that others might be having rewarding experiences from which one is absent,” represents the desire to maintain a constant connection with others and current events (Przybylski et al., 2013). With the widespread integration of social media into both personal and professional spheres, FoMO transcends individual boundaries to become a pervasive social phenomenon. Therefore, exploring the substantial role of FoMO in the negative aspects of social media is important.

Existing studies have explored various factors contributing to FoMO's development. For example, Zhao et al. (2017) categorized individual, psychological, and technological factors; Alabri (2022) synthesized the antecedents of FoMO, including belonging, group membership status, social exclusion, and social media use. However, these studies largely overlooked a critical issue: FoMO may stem from the perceived value of social media to users. In other words, it is precisely because individuals perceive social media as valuable and meaningful in their lives that they develop a fear of missing out on content or interactions. Accordingly, the first research question is as follows: How does perceived value contribute to the emergence of FoMO in the context of widespread and intensive social media exposure?

Previous research has also examined the negative effects caused by FoMO such as stress, online vulnerability, social media addiction, surface learning and fatigue (Beyens et al., 2016; Buglass et al., 2017; Ma and Liu, 2019; Rozgonjuk et al., 2019; Swiatek et al., 2021). Among these, social media fatigue is particularly notable because of its direct impact on users' willingness to engage with digital platforms. Social media fatigue refers to users' inclination to disengage from social media because of the overwhelming amount of time and energy it demands (Techopedia, 2017). As a desire to remain constantly connected with others and ongoing activities, FoMO may heighten users' experience of fatigue during social media exposure. Therefore, the second research question arises: What essential function does FoMO play in bridging users' exposure to social media and fatigue?

Although a growing body of research has established the importance of FoMO by identifying its antecedents and consequences, relatively little scholarly attention has been devoted to exploring effective mitigation strategies. In the context of constant informational temptation on social media, individuals' capacity to regulate their attention and emotions may play a critical role in shaping the intensity of FoMO. In recent years, mindfulness, conceptualized as present-moment non-judgmental awareness, has been increasingly recognized as a protective psychological practice that can alleviate anxiety and stress (Liu et al., 2020, 2022). Prior work has shown that mindfulness can promote positive engagement with social media (Yang et al., 2017; Jones et al., 2022). Accordingly, this study introduced mindfulness as a key variable and posed the third research question: Can mindfulness effectively reduce FoMO and its adverse consequences such as social media fatigue?

To address these questions, this study first reviews relevant literature and constucts a theoretical model in which FoMO functions as a mediating variable. Subsequently, data analysis is performed using the partial least squares method. Finally, this paper delves into key findings and acknowledges the limitations of the research process.

## 2 Literature review

This study examines the relationship between three phenomena: the dark side of social media, its antecedents, and a strategy for mitigation. FoMO and social media fatigue are typical manifestations of the dark side of social media. Social media exposure and perceived value serve as contributing factors, whereas mindfulness acts as a protective factor. FoMO occupies a dual role: it is itself a manifestation of the dark side of social media and simultaneously serves as a mediator linking antecedents, the mitigation strategy, and another negative outcome. This perspective not only highlights the mediating role of FoMO but also provides insights into the interconnectedness of the negative effects of social media.

### 2.1 Social media exposure and FoMO

The burgeoning popularity of social media has significantly expanded avenues for media exposure, resulting in individuals being increasingly flooded with social media content. Media exposure describes how frequently individuals encounter particular messages or types of media content (Slater, 2004). Building upon this definition, this study defines social media exposure as the level at which individuals encounter social media messages or content. “Encountering” implies simply encountering information, whether one pays attention to or remembers it (De Vreese and Neijens, 2016). Media exposure is a fundamental concept in media effects research and serves as an indispensable component of information processing (Wei and Lo, 2008). Media exposure has been studied intensively in the context of political communication. In a study of changes in public opinion during the 1980 U.S. presidential campaign, it was found that media exposure was most politically influential when prior views were weak; therefore, analyses of media effects should focus on opinions that were new or unformed (Bartels, 1993). Conversely, as the development of social media significantly expands people's unconscious media exposure, meaning that exposure occurs without a well-formed viewpoint or perspective, the media effects triggered by media exposure as an antecedent variable are equally worthy of attention.

Social media is always accessible, with the feature of “being permanently online” (Zhou, 2019). People can post messages, browse news, communicate and entertain anytime and anywhere online, which not only means that social media can meet people's needs, but also reflects a mobile Internet environment characterized by continuous connectivity. However, in contrast to social media being online at all times, human energy is limited and cannot always be online. This contradiction implies that people may always miss out on important information or situations that others have experienced, leading to FoMO.

FoMO represents a self-regulated limbic state amidst escalating information influx and is closely linked to psychological health (Przybylski et al., 2013). The inherent need for people to seek connections and belonging is a significant underlying factor driving FoMO (Rozgonjuk et al., 2019). This is evident in social media usage as people continuously check their feeds to avoid missing vital, exciting, and intriguing information. Consequently, FoMO is frequently linked to excessive smartphone use, and functions as a pivotal mechanism for comprehending problematic smartphone usage and negative affectivity (Elhai et al., 2016). Additionally, FoMO is considered a personality trait characterized by an individual's fear of missing out on something, which is particularly amplified by extensive interpersonal connections facilitated by social media platforms (Wegmann et al., 2017). Regarding individual variance, researchers tend to agree that age influences FoMO, whereas disagreement remains regarding individual differences related to gender and personality traits (Milyavskaya et al., 2018; Appel et al., 2019; Rozgonjuk et al., 2021; Ji et al., 2024). This study argues that FoMO has emerged as a widespread societal phenomenon that arises alongside pervasive and frequent exposure to social media, much like how the proliferation of television sets once gave rise to the social norm of families gathering around the TV. Accordingly, the research hypotheses and theoretical model are grounded in the contemporary context of rapid mobile internet development and individuals' increasingly ubiquitous exposure to social media.

The constant online nature of social media enables individuals to stay connected with ongoing changes in society while simultaneously increasing their susceptibility to FoMO (Coskun and Karayagiz Muslu, 2019). Different social media apps offer an increasing number of options, so people constantly check the Internet to ensure they are not missing out on what others are experiencing (Conlin et al., 2016). As a result of excessive engagement with social media platforms such as Facebook and Twitter, many users suffer from a sense of unease and anxiety when they are unable to access these apps (Wegmann et al., 2017). One study, for example, revealed a direct correlation between FoMO and social media usage (Scheinfeld and Voorhees, 2022), while another meta-analysis of 65 studies confirmed a significant positive association between FoMO and how often, how long, and how intensely people use social media (Zhang et al., 2021). Therefore, the following hypothesis is proposed that:

**H1**. Social media exposure has a significantly positive impact on FoMO.

### 2.2 Perceived value

Perceived value comes from marketing and is one of the key determinants for consumer choice, which can be conceptualized as either a unidimensional or a multidimensional construct (Sánchez-Fernández and Iniesta-Bonillo, 2007). An important and classic unidimensional concept is defined by Zeithaml (1988), after comparing value, price and quality and synthesizing different consumer expressions of value. Zeithaml (1988) described it as “consumer's overall assessment of the utility of a product based on perceptions of what is received and what is given,” where value means a balance between giving and getting. In contrast, Sheth et al. (1991) divided consumption values into five dimensions, considering it as a multidimensional structure. Sweeney and Soutar (2001) further concretized perceived value, emphasizing both the intrinsic value of an object and the user's consumption experience.

When users opt for a social media application, they engage in a process akin to choosing products and services. Therefore, perceived value, a theoretical concept extensively used in marketing, can elucidate the choice and usage behaviors of social media users. Previous research has corroborated that perceived value significantly influences users' intentions and behaviors toward using social networks (Al-Debei et al., 2013). For instance, utilitarian, social, and hedonic values can motivate users to use WeChat (Zhang et al., 2017; Pang, 2021). However, as social media platforms such as WeChat, Weibo, TikTok, and RedNote have become deeply embedded in everyday life, their usage has increasingly exhibited the characteristics of routinization and automation. In this context, the value of social media is often self-evident. The question is no longer whether users are motivated by perceived value to engage with social media, but rather how perceived value shapes their deeper psychological experiences and outcomes during habitual exposure. Accordingly, this study treats perceived value as a parallel antecedent that interacts with exposure frequency, and not as a direct antecedent of social media exposure.

Prior research suggests that the relationship itself may play a central role in shaping perceived value (Ravald and Grönroos, 1996). In a close relationship, the user's focus is not on a separate product but on the relationship as a whole and what kind of relationship and impact it maintains (Ravald and Grönroos, 1996). Therefore, from a relational perspective, users' perceived value of social media is reflected in their overall assessment of their relationship with the platform and its impact. This assessment may lead to psychological outcomes such as FoMO. Regardless of whether it is driven by the need for information, social bonding, or entertainment, if users fail to perceive the significance of their relationship with social media, they are unlikely to experience FoMO. A survey in China identified five distinct value dimensions of social media use (Zhang et al., 2022). Taken together, these considerations suggest a plausible relationship between perceived value and FoMO, leading to the following hypothesis:

**H2**. Perceived value has a significantly positive impact on FoMO.

### 2.3 Social media fatigue

Fatigue, an essential multidimensional concept in psychology and occupational health, is described as the subjective experience of unpleasant weariness (Piper et al., 1987). Fatigue has also been described as a subjective intention, with Brown (1994) defining it as a personal reluctance to continue the work at hand. As it is an emotional and personal experience, fatigue is difficult to measure and relies mostly on subjective reports, with different cultural backgrounds and languages affecting the respondents' reports of fatigue (Lewis and Wessely, 1992). As social media usage has become increasingly prevalent, attention has been drawn to social media fatigue. Based on a synthesis of prior research, social media fatigue is defined as users' subjective, self-assessed sense of weariness related to social media use (Lee et al., 2016; Jabeen et al., 2023). This reflects the overwhelming feelings that individuals feel when bombarded with massive loads of information from social networking platforms (Bright et al., 2015).

Previous studies have investigated the causes and consequences of social media fatigue. They have focused on identifying the determinants of social media fatigue, such as compulsive use, self-disclosure, information overload, FoMO, and social comparisons, and social media fatigue has been frequently incorporated into the stressor-strain-outcome framework, which measures its negative impacts, such as anxiety, academic underperformance, and depression (Dhir et al., 2018; Malik et al., 2021; Hattingh et al., 2022).

Regardless of the factors contributing to social media fatigue, the fundamental premise is extensive exposure to social media information. Especially since the COVID-19 pandemic, social media has served not only as an entertainment and social tool, but also as a significant space for professional and educational functions. Research indicates that the extent of social media engagement is a crucial factor in social media fatigue (Malik et al., 2021). Investment in time spent on social media can trigger fatigue (Baj-Rogowska, 2023). Moreover, individuals continually check social media platforms because of the fear of missing out, and this constant switching may result in feelings of exhaustion (Milyavskaya et al., 2018). Consequently, FoMO is closely linked to social media fatigue, causing people to experience greater social media fatigue (Tandon et al., 2021a; Fontes-Perryman and Spina, 2022). It was also confirmed in a study of advertising factors on social media fatigue that FoMO was the most important influencing factor, rather than the expected advertising factors. Given these arguments, this study proposes the following hypotheses:

**H3**. Social media exposure has a significantly positive impact on social media fatigue.

**H4**. FoMO has a significantly positive impact on social media fatigue.

### 2.4 Mindfulness

Mindfulness is a concept rooted in Zen and originally derived from Buddhism. After being adopted into psychology, it is characterized as “paying attention in a particular way: on purpose, in the present moment, and non-judgmentally” (Kabat-Zinn, 1994, p. 4). Non-judgment and acceptance of the current moment are two crucial elements of mindfulness (Keng et al., 2011). Prior literature has demonstrated that mindfulness alleviates anxiety (Hofmann et al., 2010) and is linked to wellbeing (Brown and Ryan, 2003). In contemporary psychology, mindfulness has been used to cope with emotional distress and undesirable behaviors (Bishop et al., 2004; Liu et al., 2025b).

Mindfulness and FoMO are opposing psychological states. An online survey of 386 undergraduate students showed that FoMO was associated with attention deficits, with higher FoMO being related to less mindful attention (Baker et al., 2016). In contrast, mindfulness emphasizes the “present moment.” When an individual is mindfully focused on the “present moment,” rumination and anticipatory thoughts are minimized, thereby reducing the sensation of FoMO (Milyavskaya et al., 2018). Mindfulness acts as a significant safeguard, especially for young people who experience high FoMO (Brailovskaia and Margraf, 2024).

Although there is limited research directly addressing social media fatigue from the perspective of mindfulness, several studies have already noted its potential to mitigate the negative effects of social media use. For example, mindfulness is acknowledged as a safeguard that buffers against the adverse effects of online activity and social media exposure (Gámez-Guadix and Calvete, 2016; Hong et al., 2021). The impact of heavy social media use on emotional exhaustion was moderated by mindfulness, with employees experiencing lower social media fatigue at higher levels of mindfulness (Charoensukmongkol, 2016). All these studies suggest that mindfulness not only helps individuals become aware of their current state but also facilitates adaptive behavioral responses through enhanced self-regulation (Damico and Krutka, 2018), which may be the mechanism by which mindfulness exerts its beneficial effects. The validation of the Flow Ergonomics scale further demonstrates the link between mindfulness and psychological outcomes (Chiou et al., 2022). Therefore, it is justifiable to assume that mindfulness can mitigate social media fatigue. Given the above discussion, the following hypotheses are proposed:

**H5**. Mindfulness has a significantly negative impact on FoMO.

**H6**. Mindfulness has a significantly negative impact on social media fatigue.

**Figure 1:**
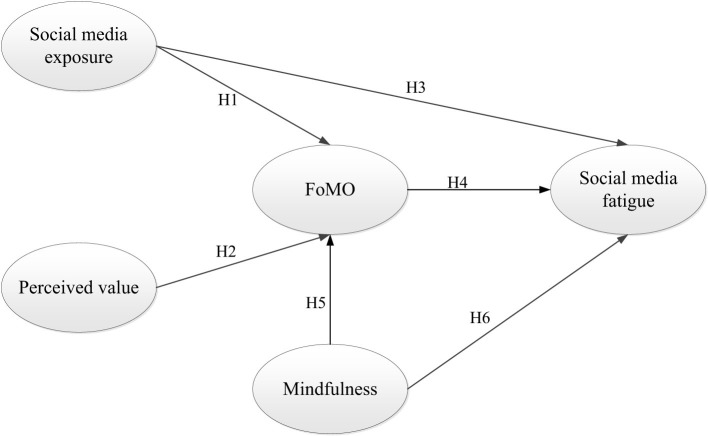
Research model.

In conclusion, aiming to analyze the causes, importance, and targeted inhibition method of FoMO, an integrated model (Figure 1) is established based on previous literature, including the constructs of social media exposure, perceived value, mindfulness, FoMO, and social media fatigue.

## 3 Research method

### 3.1 Sample

The present study surveyed social media users aged 18–45 years based on both theoretical and practical considerations. Theoretically, prior research has established that FoMO is age-related, with younger individuals typically reporting higher levels of FoMO (Przybylski et al., 2013). According to China's Middle- and Long-term Youth Development Plan (2016–2025; China Daily, 2017), the age range for youth is 14–35 years, which provides a foundational reference point for the sampling frame. From a practical standpoint, frequent and intensive social media engagement in China extends beyond narrowly defined youth cohorts. Individuals in their late 30s and 40s are also highly active users, especially on platforms such as WeChat, which serve not only as strong-tie social networks, but also as tools for professional communication and everyday coordination. According to t*he 49th Statistical Report on China's Internet Development* by CNNIC (2022), users aged 20–29, 30–39, and 40–49 years accounted for 17.3%, 19.9%, and 18.4% of all Internet users, respectively, far surpassing other age groups. While the report reflects general Internet access rather than specific platform preferences, it clearly highlights the significant participation of users around their 40s in the digital landscape. Therefore, while the theoretical focus was on younger users, the sample was extended to age 45 to better reflect the actual demographic composition of active social media users in China. This age range allowed this study to capture a wider spectrum of FoMO-related experiences, covering both younger and early middle-aged users who are actively involved in digital social interactions.

Before the formal questionnaire survey, this study conducted interviews and pre-surveys with eight social media users and modified the questionnaire based on the feedback received. Formal and anonymous questionnaires were distributed and collected through social media platforms, namely QQ and WeChat, in August and September 2022. To increase the effectiveness of respondents' answers, each questionnaire was accompanied by a red packet of 1 RMB. Eventually, 406 valid questionnaires were obtained, and the sample size conformed to the recommended sample size of 250–500 for structural equation modeling suggested by Schumacker and Lomax (2016). The demographic characteristics of the participants are shown in Table 1.

**Table 1:** Respondent demographics and social media use.

**Items**	**Type**	**Frequency**	**Percent (%)**
Gender	Male	204	50.2
	Female	202	49.8
Age	18–25	135	33.3
	26–30	97	23.9
	31–40	125	30.8
	41–45	49	12.1
Education	High school or below	27	6.7
	Undergraduate	213	52.5
	Master's degree	128	31.5
	Doctoral degree	38	9.4
Time of use per day	30 min and less	10	2.5
	More than 30 min to 2 h	111	27.3
	Over 2–4 h	127	31.3
	Over 4–6 h	86	21.2
	Over 6 h	72	17.7
Increase in frequency of social media use since COVID-19	Not increased	122	30.0
	25% and below	68	16.7
	More than 25%−50%	108	26.6
	More than 50%−75%	62	15.3
	More than 75%	46	11.3

### 3.2 Measures

The variable measurements in this study fully absorbed and drew on previous research and adjusted existing scale questions to consider the situation of Chinese people's use of social media. All constructs were measured using a Five-point Likert scale. For social media exposure (SME), the response scale ranged from 1 = “Never” to 5 = “Very often.” For all other constructs, the scale ranged from 1 = “Not at all true of me” to 5 = “Extremely true of me.”

Social media exposure was measured using respondents' self-reports, as prior research on this construct has relied primarily on self-report measures (De Vreese and Neijens, 2016). To assess perceived value, five items were adapted from previous literature and the PERVAL scale (Sheth et al., 1991; Sweeney and Soutar, 2001; Zhang et al., 2017). To assess FoMO, this study primarily employed the FoMO scale developed by Przybylski et al. (2013). Although the scale was not originally designed for online contexts, this in itself highlights that, in an era dominated by digital technology, FoMO has become a pervasive phenomenon in which the boundaries between online and offline environments are increasingly blurred. Building on prior studies (Wegmann et al., 2017; Song et al., 2017), the FoMO scale was adapted to the Chinese social media context, resulting in a revised seven-item measure used in this study. To assess social media fatigue, four items were adapted from Bright et al. (2015) and Lee et al. (2016) to measure psychological fatigue in the context of social media use. Finally, to assess mindfulness, it is important to acknowledge its inherently abstract nature. As noted earlier, during the pre-survey phase, participants reported confusion with several items from established mindfulness scales (e.g., “I will notice some of my feelings…”), which appeared too vague or introspective for general respondents. Therefore, based on the MAAS and FFMQ mindfulness scales (Brown and Ryan, 2003; Deng et al., 2011), and considering the Chinese cultural context, this study adopted five items that were more concrete and closely connected to daily life experiences. These items were intended to capture the essence of dispositional mindfulness: being purposeful, present, and non-judgmental (Kabat-Zinn, 1994, p. 4). All constructs and measurement items are detailed in Table 2.

**Table 2:** Constructs and items.

**Construct**	**Item**	**Measuring project**	**Literature**
Social media exposure	SME1	Frequency of daily engagement with social media platforms	Adapted from Wei and Lo (2008), Gao et al. (2020)
	SME2	Daily occurrence of social media chats	
	SME3	Frequency of weekly engagement with social media platforms	
Perceived value	PV1	Social media stimulates my curiosity	Adapted from Sheth et al. (1991), Sweeney and Soutar (2001), Zhang et al. (2017)
	PV2	Social media can bring me new knowledge	
	PV3	Social media is useful	
	PV4	Social media is helpful	
	PV5	Social media is a joy of life	
FoMO	FoMO1	I regularly monitor social media to stay updated and not miss anything	Adapted from Przybylski et al. (2013), Wegmann et al. (2017), Song et al. (2017)
	FoMO2	When I am having a good time, sharing on social media is important to me (e.g. send it to a moment or a tweet)	
	FoMO3	During holidays, I will stay updated on my friends' activities through social media	
	FoMO4	During holidays, I will continue to pay close attention to hot news through social media	
	FoMO5	If there is an update on my social media, I want to check on it immediately	
	FoMO6	Using social media is something I can't live without	
	FoMO7	Being without social media for a few days leaves me feeling lost and uncomfortable	
Social media fatigue	SMF1	I am often drowning in a flood of social media information	Adapted from Bright et al. (2015), Lee et al. (2016)
	SMF2	Following social media use, I struggle to focus during my leisure time	
	SMF3	The excessive information flow on social media triggers anxiety for me	
	SMF4	After spending time on social media, I experienced fatigue	
Mindfulness	MIND1	I get distracted easily	Adapted from Brown and Ryan (2003), Deng et al. (2011)
	MIND2	I find I cannot focus when doing things	
	MIND3	I struggle to articulate my thoughts	
	MIND4	I often find my mind dwelling on the past or the future	
	MIND5	I blame myself for having irrational or inappropriate emotions	

## 4 Data analysis

This study employed partial least squares structural equation modeling (PLS-SEM) for analysis. Compared to covariance-based SEM (CB-SEM), PLS-SEM is better suited for prediction and theory development and does not require raw data to meet normality assumptions, thereby providing more robust results (Hair et al., 2011). As the objective of this study was to explore FoMO's significant role in the negative aspects of social media and to identify an alleviating factor, and since data collection was conducted through an online questionnaire, PLS-SEM was considered more appropriate. Both the outer and inner models were assessed using SPSS 26.0 and SmartPLS 4.0.

### 4.1 Outer model analysis

Before assessing the significant relationships between the constructs, it was essential to test the consistency and validity of the outer model (Fornell and Larcker, 1981). Factor loadings, composite reliability (CR), Cronbach's alpha, and average variance extracted (AVE) are indicators of reliability and convergent validity (Urbach and Ahlemann, 2010). Therefore, this study initially assessed each construct to ensure internal consistency and convergent validity. As depicted in Table 3, each construct demonstrated Cronbach's α values exceeding 0.7, CR values surpassing 0.8, and AVE values greater than 0.5, indicating robust reliability and convergent validity (Urbach and Ahlemann, 2010).

**Table 3:** Reliability and convergent validity.

**Construct**	**Item**	**Mean**	**Factor loading**	**Cronbach's a**	**CR**	**AVE**
Social media exposure	SME1	4.392	0.822	0.728	0.845	0.646
	SME2	4.007	0.723			
	SME3	4.443	0.860			
Perceived value	PV1	3.291	0.787	0.895	0.923	0.706
	PV2	3.480	0.826			
	PV3	3.515	0.842			
	PV4	3.557	0.891			
	PV5	3.655	0.852			
FoMO	FoMO1	3.495	0.705	0.866	0.897	0.555
	FoMO2	2.926	0.714			
	FoMO3	3.049	0.786			
	FoMO4	3.438	0.767			
	FoMO5	3.096	0.755			
	FoMO6	3.618	0.731			
	FoMO7	3.106	0.754			
Mindfulness	MIND1	3.037	0.863	0.847	0.892	0.624
	MIND2	3.076	0.872			
	MIND3	3.387	0.766			
	MIND4	3.116	0.747			
	MIND5	2.929	0.685			
Social media fatigue	SMF1	3.308	0.704	0.825	0.883	0.655
	SMF2	3.002	0.844			
	SMF3	2.818	0.862			
	SMF4	2.978	0.818			

Next, this study assessed discriminant validity using the Fornell-Larcker criterion, followed by validation using the heterotrait-monotrait ratio (HTMT). As shown in Table 4, for each construct, the square root of its AVE exceeds its correlations with other constructs, confirming good discriminant validity (Fornell and Larcker, 1981). According to the HTMT method presented in Table 5, the HTMT values between all constructs were below 0.85, thus strengthening the evidence of good discriminant validity (Henseler et al., 2015). Therefore, the model demonstrated satisfactory discriminant validity.

**Table 4:** Discriminant validity—Fornell-Larcker criterion.

**Construct**	**MIND**	**FoMO**	**PV**	**SME**	**SMF**
MIND	0.790				
FoMO	−0.437	0.745			
PV	−0.319	0.665	0.840		
SME	−0.188	0.370	0.339	0.804	
SMF	−0.622	0.439	0.242	0.218	0.810

SME, Social media exposure; PV, Perceived value; FoMO, Fear of missing out; SMF, Social media fatigue; MIND, Mindfulness.

**Table 5:** Discriminant validity—HTMT.

**Construct**	**MIND**	**FoMO**	**PV**	**SME**	**SMF**
MIND					
FoMO	0.514				
PV	0.364	0.748			
SME	0.224	0.459	0.409		
SMF	0.717	0.517	0.281	0.277	

SME, Social media exposure; PV, Perceived value; FoMO, Fear of missing out; SMF, Social media fatigue; MIND, Mindfulness.

### 4.2 Inner model analysis

**Figure 2:**
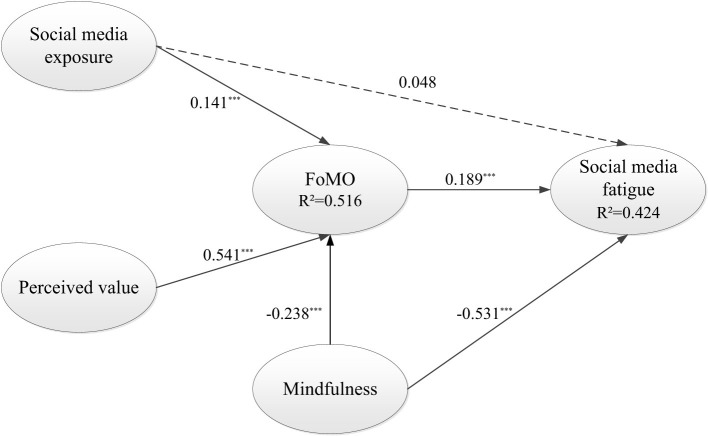
Results of the research model. ***p < 0.001.

The bootstrapping procedure in SmartPLS, utilizing 5,000 subsamples, was employed to analyze path coefficients and indirect effects within the inner model. The pathway analysis and hypothesis-testing outcomes for the research model are depicted in Figure 2 and outlined in Table 6.

**Table 6:** Summary of structural model results.

**Hypothesis**	**Path coefficient**	***t*-Value**	**Result**
H1 SME → FoMO	0.141	3.926^***^	Supported
H2 PV → FoMO	0.541	14.220^***^	Supported
H3 SME → SMF	0.048	1.235	Not supported
H4 FoMO → SMF	0.189	3.617^***^	Supported
H5 MIND → FoMO	−0.238	5.661^***^	Supported
H6 MIND → SMF	−0.531	11.624^***^	Supported

SME, Social media exposure; PV, Perceived value; FoMO, Fear of missing out; SMF, Social media fatigue; MIND, Mindfulness.

^***^p < 0.001.

First, all hypotheses were supported, except for the effect of social media exposure on social media fatigue, which was not significant. Second, in examining predictors of FoMO, social media exposure (β = 0.141, *p* < 0.001) and perceived value (β = 0.541, *p* < 0.001) both had significant positive effects, with perceived value demonstrating a notably stronger effect. Mindfulness exhibited a significant negative effect on FoMO (β = −0.238, *p* < 0.001), indicating its role in alleviating FoMO. Third, regarding social media fatigue, both FoMO and mindfulness significantly influenced it, with FoMO exerting a positive effect (β = 0.189, *p* < 0.001) and mindfulness a negative effect (β = −0.531, *p* < 0.001).

The R^2^ value indicates the model's explanatory power for the variance in the dependent variable. As shown in Figure 2, the R^2^ values for FoMO and social media fatigue are 0.516 and 0.424, respectively. (Chin 1998) suggests that an R^2^ value around 0.67 indicates substantial explanatory power, while a value near 0.33 is considered moderate. Thus, the current model demonstrates good explanatory capability. Moreover, PLSpredict provides a means to evaluate the out-of-sample predictive power (Shmueli et al., 2019). As shown in Table 7, all Q^2^predict values are above 0, and the RMSE values from PLS-SEM analysis are all lower than those from the linear regression model (LM) benchmark, indicating that the model has good predictive power (Shmueli et al., 2019).

**Table 7:** PLSpredict results.

**Item**	**Q^2^ predict**	**PLS-SEM RMSE**	**LM RMSE**	**Difference**
FoMO1	0.244	0.890	0.910	−0.020
FoMO2	0.251	0.980	0.984	−0.004
FoMO3	0.306	0.913	0.925	−0.012
FoMO4	0.398	0.806	0.813	−0.007
FoMO5	0.240	0.893	0.919	−0.026
FoMO6	0.287	0.886	0.891	−0.005
FoMO7	0.217	1.050	1.071	−0.021
SMF1	0.093	1.012	1.027	−0.015
SMF2	0.335	0.891	0.901	−0.010
SMF3	0.297	0.900	0.917	−0.017
SMF4	0.245	0.944	0.953	−0.009

FoMO, Fear of missing out; SMF, Social media fatigue.

Regarding the analysis of indirect effects, as presented in Table 8, all indirect effects were supported, confirming the mediating role of FoMO. First, social media exposure (β = 0.027, *p* < 0.01) and perceived value (β = 0.102, *p* < 0.001) exhibited significant positive indirect effects on social media fatigue through FoMO, while mindfulness (β = −0.045, *p* < 0.01) demonstrated a significant negative indirect effect. Second, although social media exposure did not have a significant direct effect on social media fatigue, its indirect effect through FoMO was significant, highlighting FoMO's important mediating function.

**Table 8:** Analysis of indirect effects.

**Relationship**	**Path coefficient**	***t*-Value**	**Result**
SME → FoMO → SMF	0.027	2.710^**^	Supported
PV → FoMO → SMF	0.102	3.580^***^	Supported
MIND → FoMO → SMF	−0.045	2.836^**^	Supported

SME, Social media exposure; PV, Perceived value; FoMO, Fear of missing out; SMF, Social media fatigue; MIND, Mindfulness.

^**^p < 0.01; ^***^p < 0.001.

**Figure 3:**
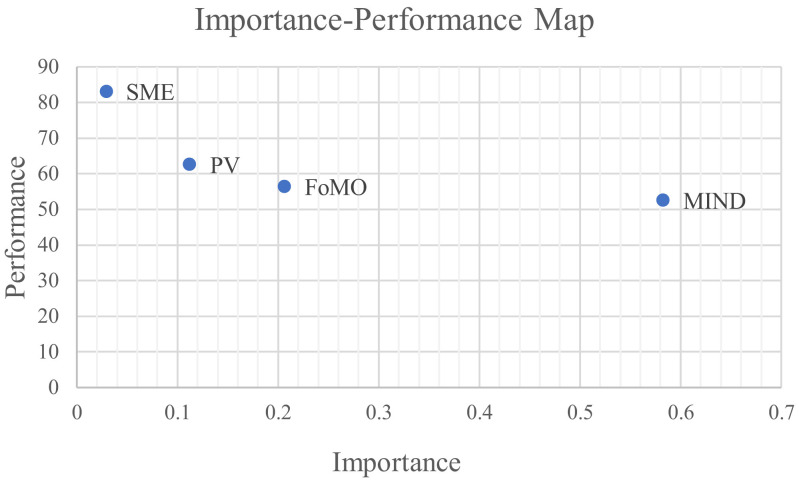
Importance-performance map of construct SMF.

In addition, researchers are encouraged to complement PLS-SEM results with Importance-Performance Map Analysis (IPMA), which allows for prioritization of managerial actions based on both the importance and performance of target construct (Ringle and Sarstedt, 2016). As shown in Table 9 and Figure 3, the IPMA results reveal that for the target construct social media fatigue, its two direct antecedents, mindfulness and FoMO, exhibit the highest importance values (0.582 and 0.206, respectively), substantially exceeding those of other predictors. However, their performance scores are relatively low, at 52.618 for mindfulness and 56.484 for FoMO, suggesting significant potential for improvement. Consequently, practical interventions should prioritize enhancing the performance of mindfulness, given its dominant influence, while also addressing FoMO to effectively mitigate social media fatigue.

**Table 9:** Importance-performance map (IPMA) results for construct SMF.

**Construct**	**Importance**	**Performance**
SME	0.029	83.173
PV	0.112	62.610
FoMO	0.206	56.484
MIND	0.582	52.618

SME, Social media exposure; PV, Perceived value; FoMO, Fear of missing out; SMF, Social media fatigue; MIND, Mindfulness.

Importance values are reported as absolute values.

## 5 Discussion and conclusion

To explore the pivotal role of FoMO in the negative impacts of social media and identify an effective mitigation strategy, this study examined the relationships between social media exposure, perceived value, mindfulness, FoMO, and social media fatigue, focusing particularly on FoMO's mediating function and mindfulness's buffering effect in reducing FoMO and social media fatigue.

First, perceived value served as an important antecedent of FoMO and appeared to be the stronger predictor in the model. This shows that users' overall evaluations of their relationships with social media platforms can be an important contributor to the development of FoMO. When individuals perceive social media as more meaningful and valuable, they are more likely to worry about missing content or social interactions. Conversely, when the perceived value is low, the level of FoMO tends to decrease. Given the multidimensional nature of perceived value, different social media platforms may elicit distinct value perceptions (Zhang et al., 2022). For example, WeChat, as a prototypical “strong-tie” platform, emphasizes sustained connections with close social ties, so users may experience FoMO out of concern regarding missing important updates related to friends and family members. In contrast, RedNote, a lifestyle-oriented interest community, creates user value through personalized recommendations and everyday-life guidance (Jiang and Pang, 2025), potentially reinforcing FoMO differently. Thus, this study identifies a significant association between perceived value and FoMO, revealing not only the important role of perceived value in the formation of FoMO but also the profound significance of social media in shaping users' relationships and behaviors. When perceived value and social media exposure are jointly considered antecedents of FoMO, the formation of FoMO as a normalized socio-psychological phenomenon becomes more intelligible. In other words, in the context of the widespread and routinized use of social media, users' perceptions of value can be a critical driver of FoMO.

Second, the results indicate that FoMO is itself a manifestation of the dark side of social media while simultaneously functioning as a mediating mechanism in the development of social media fatigue. In this study's model, social media exposure, perceived value, and mindfulness influenced social media fatigue through FoMO. Specifically, both social media exposure and perceived value significantly exacerbated fatigue through heightened FoMO, whereas mindfulness alleviated fatigue by reducing FoMO levels. This finding aligns with previous studies that have identified FoMO as a key mediator (Wang et al., 2018; Swiatek et al., 2021). Notably, this study also found that social media exposure does not have a direct effect on social media fatigue; however, its impact becomes significant through the mediating role of FoMO. This suggests that FoMO can serve as a conduit that intensifies other adverse outcomes, thereby fostering a vicious cycle (Oberst et al., 2017). To further explain this mediating role, it is important to consider how FoMO contributes to social media fatigue. As Salo et al. (2018) noted, the dark side of social media consists of a continuum of overlapping shades from gray to black, and FoMO may represent a critical link within this spectrum. By increasing the cognitive load and inducing ongoing attentional distraction and anxiety, FoMO may convert social media exposure into a burdensome experience marked by information overload and psychological fatigue.

Third, mindfulness showed a significant negative association with both FoMO and social media fatigue, indicating its efficacy in alleviating these issues and supporting prior findings on its constructive role in mitigating the adverse repercussions of social media (Apaolaza et al., 2019; Hong et al., 2021). Mindfulness sustains focused attention while remaining aware of both internal and external experiences (Liu et al., 2023, 2025a), thereby reducing distraction (Jain et al., 2007) and, in turn, alleviating FoMO and social media fatigue. This finding regarding mindfulness provides a new perspective for understanding FoMO: rather than seeking to eliminate FoMO, individuals can adopt a mindful approach to social media use to reduce both FoMO and its adverse consequences. Specifically, being consciously present while engaging with the content—as opposed to passively revisiting or compulsively monitoring updates—can serve as a buffer against fatigue. These findings underscore that, while FoMO is a key driver of social media's negative impacts, it should not be treated as an insurmountable force. As FoMO becomes a normalized by-product of digital culture, the focus should shift from eradication to regulation, with mindfulness offering a promising strategy.

Notably, this study did not find a significant direct association between social media exposure and fatigue. A plausible explanation is the mediating role of FoMO, which may obscure a direct relationship by serving as a conduit through which social media exposure influences fatigue. Moreover, as FoMO often compels individuals to check social media repeatedly, it may reinforce exposure intensity, thereby creating a self-reinforcing cycle that further complicates direct linkage. Additionally, the reliance on self-reported measures and use of a cross-sectional design may constrain the ability to detect causal effects.

Theoretically, the FoMO mediation model developed in this study has several implications. First, the findings highlight the pivotal mediating role of FoMO in the negative outcomes of social media use, revealing that phenomena such as FoMO and social media fatigue are not isolated occurrences but rather part of a dynamic and interrelated psychological system. Second, this study identifies perceived value as an important antecedent of FoMO, providing a conceptual basis for future research on this relationship. Finally, by incorporating mindfulness as a key variable, this research extends its theoretical relevance to the domain of digital media use, highlighting its potential as a protective factor within online environments.

Practically, the significant buffering effect of mindfulness on both FoMO and social media fatigue underscores the potential of internal psychological resources in addressing the negative effects induced by persistent digital engagement. In this respect, mindfulness emerges not only as a theoretically meaningful construct but also as a promising intervention strategy in the digital era, as also evidenced in design-oriented applications integrating mindfulness or related psychological resources to alleviate negative psychological states (Chiou et al., 2021; Liu et al., 2021). The IPMA results further suggest that interventions should prioritize enhancing mindfulness to more effectively mitigate social media fatigue. Since the COVID-19 pandemic, social media platforms such as WeChat have evolved into indispensable tools for numerous individuals, fostering a pervasive sense of constant online connectivity. In the survey of this study, nearly 40% of the respondents reported spending over 4 h on social media daily, with more than half indicating an increase in usage of more than 25% after the pandemic. In this context, the findings of this study imply that enhancing individuals' levels of mindfulness may promote greater self-awareness and emotional regulation, thereby strengthening their psychological resilience and wellbeing (Brown and Ryan, 2003). Furthermore, this study deepens our understanding of FoMO as a normalized psychosocial phenomenon. FoMO should not be treated as a pathology to be suppressed but rather as a psychological signal to be understood and managed. By clarifying its triggers and buffering mechanisms, such as perceived value and mindfulness, this study offers practical guidance for fostering reflective, adaptive, and psychologically sustainable media engagement.

## 6 Limitations and future directions

This study has certain limitations. First, although the proposed mediation model received statistical support, its cross-sectional design limited its ability to draw causal inferences regarding the relationships among the variables. Therefore, future research should use longitudinal or experimental designs to rigorously test the hypothesized pathways and examine the temporal dynamics of these psychological processes. Second, social media comprise a diverse ecosystem of platforms, each with distinct affordances and interactional norms. Owing to practical constraints, this study did not differentiate FoMO experiences across specific applications such as WeChat, Weibo, TikTok, or RedNote. Future studies should consider platform-specific analyses to better understand how varying design features and user cultures affect psychological responses. Finally, the sample comprised general social media users who had not received formal mindfulness training. A simplified five-item scale was used to enhance accessibility and reflect cultural sensitivity. While pragmatic, this approach may have constrained the construct validity of the mindfulness assessment. Given this, future research should employ more comprehensive instruments or experimental manipulations of mindfulness to capture its psychological function within social media contexts more precisely.

## Data Availability

The original contributions presented in the study are included in the article/supplementary material, further inquiries can be directed to the corresponding author.
